# Low-Dose Urinary Human Chorionic Gonadotropin Is Effective for Oocyte Maturation in *In Vitro* Fertilization/ Intracytoplasmic Sperm Injection Cycles Independent of Body Mass Index

**DOI:** 10.22074/ijfs.2016.5145

**Published:** 2016-11-11

**Authors:** Luis R. Hoyos, Sana Khan, Jing Dai, Manvinder Singh, Michael P. Diamond, Elizabeth E. Puscheck, Awoniyi O. Awonuga

**Affiliations:** 1. Department of Obstetrics and Gynecology, Wayne State University School of Medicine, Detroit Medical Center, Detroit, MI, USA; 2. Division of Reproductive Endocrinology and Infertility, Department of Obstetrics and Gynecology, Wayne State University School of Medicine, Detroit Medical Center, Detroit, MI, USA; 3. C.S. Mott Center for Human Growth and Development, Wayne State University School of Medicine, Detroit Medical Center, Detroit, MI, USA; 4. Division of Reproductive Endocrinology and Infertility, Department of Obstetrics and Gynecology, Augusta University, Augusta, GA, USA

**Keywords:** Body Mass Index, Urinary Human Chorionic Gonadotropin, *In Vitro* Fertilization, Pregnancy Rate, Live Birth Rate

## Abstract

**Background:**

Currently, there is no agreement on the optimal urinary derived human chorionic gonadotropin (u-hCG) dose requirement for initiating final oocyte maturation prior to oocyte collection in *in vitro* fertilization (IVF), but doses that range from 2500- 15000 IU have been used. We intended to determine whether low dose u-hCG was effective for oocyte maturation in IVF/intracytoplasmic sperm injection (ICSI) cycles independent of body mass index (BMI).

**Materials and Methods:**

We retrospectively evaluated a cohort of 295 women who underwent their first IVF/ICSI cycles between January 2003 and December 2010 at the Division of Reproductive Endocrinology and Infertility, Wayne State University, Detroit, MI, USA. Treatment cycles were divided into 3 groups based on BMI (kg/ m^2^): <25 (n=136), 25- <30 (n=84), and ≥30 (n=75) women. Patients received 5000, 10000 or 15000 IU u-hCG for final maturation prior to oocyte collection. The primary outcome was clinical pregnancy rates (CPRs) and secondary outcome was live birth rates (LBRs).

**Results:**

Only maternal age negatively impacted (P<0.001) CPR [odds ratio (OR=0.85, confidence interval (CI: 0.79-0.91)] and LBR (OR=0.84, CI: 0.78-0.90).

**Conclusion:**

Administration of lower dose u-hCG was effective for oocyte maturation in IVF and did not affect the CPRs and LBRs irrespective of BMI. Women’s BMI need not be taken into consideration in choosing the appropriate dose of u-hCG for final oocyte maturation prior to oocyte collection in IVF. Only maternal age at the time of IVF negatively influenced CPRs and LBRs in this study.

## Introduction

Extremes in body weight have a significant impact on overall health and fertility (1). However, lower fertility rates in obese women may not be exclusively related to ovulatory dysfunction (2-5) as there is evidence to suggest that excess weight and its associated hyperglycemia increase intrafollicular glucose levels (6) that may adversely affect oocyte (7), embryo (8, 9), and endometrial (10, 11) quality. The follicular fluid composition of patients who undergo assisted reproductive technology (ART) with different body mass indexes (BMIs, kg/m^2^) has been analyzed and compared to oocyte collection, embryo development, and pregnancy rates (PRs) in a study by Robker et al. (12). The authors reported a significant effect on oocyte quality by increased BMI with less oocytes collected and less embryos produced from obese patients (BMI≥30). This has led some (13), but not all (14), experts to suggest that BMI should influence ART protocols.

Urinary derived human chorionic gonadotropin (u-hCG) is used instead of luteinizing hormone (LH) to trigger final oocyte maturation prior to oocyte collection in ART because of its long half-life and stronger impact on the follicle. The hCG concentrations in serum that reach the follicles should be at a level capable of initiating meiosis and triggering the release of the cumulus-oocyte complex into the follicular fluid. While some authorities (15, 16), including our group (17), have reported a significant negative correlation between BMI and serum hCG levels post u-hCG administration, others report no difference in these levels in relation to BMI (18). Currently there is no agreement on the optimal u-hCG dose requirements for initiating final oocyte maturation prior to oocyte collection in *in vitro* fertilization (IVF); doses that range from 2500-15000 IU have been used (17, 19, 20) and often depended on the number of follicles that developed, the peak serum estradiol (E_2_) level, and the perceived risk of ovarian hyperstimulation syndrome (OHSS). 

These studies did not specifically include obese patients. The present manuscript has sought to determine the effectiveness of lower dose u-hCG (5000 IU) compared to higher doses (10000 or 15000 IU) for final oocyte maturation in IVF/ intracytoplasmic sperm injection (ICSI) cycles independent of BMI. Our null hypothesis was that the commonly administered lower dose of u-hCG (5000 IU) compared with 10000 and 15000 IU would not adversely affect clinical pregnancy rates (CPRs) and live birth rates (LBRs) across the different BMI strata [i.e., <25, 25 - <30 (overweight), and ≥30 (obese)]. The results of this study might provide a better understanding of the effect of BMI on lower u-hCG doses when used in lieu of the LH surge.

## Materials and Methods

After obtaining approval for this retrospective cohort study from the Institutional Review Board of Wayne State University, we conducted a study of 295 women who had their first IVF/ICSI cycles (out of a total 467 cycles) in our institution between January 2003 and December 2010. Reasons for exclusion of cycles from analysis included: cycles other than the first IVF/ICSI cycles (n=114), missing data such that BMI could not be calculated (n=24), hCG levels that were not drawn 12-14 hours after administration of the u-hCG dose (n=17), and cycles in which booster u-hCG doses were given after the initial trigger u-hCG dose (n=17). Booster doses of u-hCG were administered when the patient’s 12-14 hour levels were perceived to be low by the care provider. All 295 women had oral contraceptive pill administration following which they underwent ovarian stimulation with gonadotropins (Gn) - mainly recombinant follicle-stimulating hormone (FSH) and preparations that contained equal amounts of FSH and LH, and gonadotropin releasing hormone (GnRH) agonists [6 (2.0%)] or antagonists [289 (98.0%)]. 

We administered u-hCG when a minimum of two follicles with a mean diameter >18 mm were identified on transvaginal ultrasound and there were at least four follicles between 16-20 mm. Patients received varying doses of u-hCG (5000 IU, 10000 IU, or 15000 IU) administered intramuscularly depending on the number of follicles that developed and their peak E_2_ levels. The lower dose (5000 IU) was often administered when there was a higher perceived risk of OHSS (E_2_ ≥3000 pg/mL). Poor responders who barely attained our minimum criteria for proceeding to oocyte collection (4 follicles between 16-20 mm and appropriate E_2_ levels for the size and number of follicles) received the 15000 IU u-hCG dose. The patient’s BMI was not a factor in determining the doses of u-hCG given to trigger ovulation in any of these cases. Blood for serum hCG levels were drawn 12-14 hours after u-hCG administration, mainly to ascertain that patients appro- priately self-administered the drug. This time point was based on a study by Chan et al. (21) where they reported that serum hCG levels peaked at 12 hours after the injection and decreased thereafter. Oocyte collection was performed 36 hours after the hCG trigger. Embryo transfer was performed on days 2 to 5 after oocyte collection. We defined the implantation rate as the number of gestation sacs per embryo transferred, while CPR was defined as number of cycles with intrauterine gestational sac(s) with fetal heart pulsation at 6-8 weeks from the day of transfer. Luteal support was with intramuscular 100 mg progesterone in oil that began on the evening of oocyte collection. When pregnant, patients continued progesterone supplementation until 12 weeks of pregnancy (10 weeks after retrieval). Treatment cycles were divided into 3 groups based on BMI (kg/m^2^): <25 (n=136), 25- <30 (overweight, n=84) and ≥30 (obese, n=75).

Data were analyzed using SPSS version 22.0 and presented as mean ± SE. Most of our independent variables did not have a normal distribution. Hence, in order to satisfy the normality assumptions of ANOVA, we used log algorithm to transform infertility duration, the total dose of Gn used for superovulation, and hCG levels at 12-14 hours. The baseline FSH (drawn on day 3 of the menstrual cycle), the total number of follicles that developed, follicles ≥14 mm and E_2_ on the day of u-hCG trigger, number of mature oocytes and hence subjected to ICSI, number of oocytes that fertilized, and endometrial thickness on the day u-hCG trigger was administered were transformed using square root algorithm. Patients’ ages, the number of days patients received Gn stimulation, and the number of embryos transferred had normal distributions and were not transformed. 

First, we performed a one-way ANOVA to compare the mean dose of u-hCG administered in relation to BMI categories as well as the mean BMI in the different u-hCG doses administered. Next, we performed two-way ANOVA to determine the main effects and interaction between the independent variables (demographics, superovulation parameters, dose of u-hCG administered, and BMI) and the dependent variables (CPRs and LBRs). Tukey’s post hoc tests were performed for mean separation following the detection of a significant main effect. Gravidity, parity, and antral follicle count (AFC) were analyzed using non-parametric Friedman’s two-way ANOVA. Log linear analysis was conducted to examine the association among diagnosis, BMI, and u-hCG categories. 

We used binary logistic regressions to model CPRs and LBRs with forward stepwise selection according to the likelihood ratio method based on inclusion/exclusion criteria of P≤0.05/P>0.10. The BMI, u-hCG doses given for ovulation trigger along with all significant factors in Tables 1, 2 and 3 were included in the model. Mother’s age was also included based on significant correlations with both CPRs (r=-0.28 and P<0.001) and LBRs (r=-0.028 and P<0.0001). Statistical significance was defined as P<0.05.

## Results

Initially we conducted log linear analysis to examine the association among etiology of infertility, BMI, and u-hCG categories. No association was detected. Etiologies of infertility were thus presented as frequencies and percentages for the whole population. Infertility causes included: sperm dysfunction in 141 (47.8%), tubal disease in 71 (24.1%), ovulatory dysfunction in 48 (16.3%), and unexplained in 18 (6.1%) patients. The remaining 17 (5.8%) patients had other causes for their infertility.

Next, we conducted two-way ANOVAs that contained demographic variables because no interaction between BMI and HCG was detected using a full model. Patients’ demographic variables in relation to BMI and u-hCG dose categories are shown in Table 1. Obese women had a significantly higher infertility duration compared to overweight individuals and those with BMI <25. However, a significantly lower baseline day 3 FSH existed in obese women compared to those with BMI <25 and overweight women. These values were not different between the latter two groups of women. No differences in maternal age, gravidity, parity, and AFC existed in relation to weight distribution. Similar to weight distribution, we observed significantly lower baseline FSH in those who received 5000 and 15000 IU u-hCG for ovulation trigger compared with those who received 10000 IU. No difference in baseline FSH existed between the former two groups. However, there was a significantly higher mean AFC in those who received 5000 IU u-hCG compared with patients who received 10000 and 15000 IU. We observed no difference in the latter two groups in terms of mean AFC (Table 1). 

**Table 1:** Demographic variables in relation to BMI categories and u-hCG dosages (n=295)


Variable		BMI				u-hCG		
	<25	25 - <30	≥30	P value	5000	10000	15000	P value

Age (Y)	33.8 ± 0.4	34.1 ± 0.5	33.9 ± 0.6	NS	33.1 ± 0.8	34.2 ± 0.4	33.5 ± 0.5	NS
	n=136	n=84	n=75		n=39	n=193	n=63	
Infertility duration (months)	38.1 ± 2.5	38.3 ± 3.4	50.0 ± 5.5	0.09	34.6 ± 5.8	42.9 ± 2.5	39.8 ± 5.0	NS
	n=109	n=66	n=64		n=32	n=176	n=31	
Day 3 FSH (mIU/L)	7.2 ± 0.2^a^	7.6 ± 0.5^a^	6.4 ± 0.3^b^	0.031	6.4 ± 0.3^b^	7.4 ± 0.2^a^	6.8 ± 0.6^b^	0.003
	n=128	n=81	n=72		n=36	n=190	n=54	
Gravidity	0.7 ± 0.1	1.2 ± 0.3	1.2 ± 0.2	NS	0.8 ± 0.2	1.0 ± 0.1	1.1 ± 0.3	NS
	n=136	n=84	n=75		n=39	n=193	n=63	
Parity	0.3 ± 0.1	0.4 ± 0.1	0.4 ± 0.1	NS	0.4 ± 0.1	0.4 ± 0.1	0.3 ± 0.1	NS
	n=136	n=84	n=75		n=39	n=193	n=63	
AFC	13.5 ± 0.8	12.5 ± 0.9	15.4 ± 1.2	NS	18.8 ± 1.9^a^	13.1 ± 0.6^b^	12.0 ± 1.6^b^	0.006
	n=107	n=74	n=65		n=31	n=187	n=28	


Results are means ± SE from two-way ANOVAs presented with Tukey post hoc analysis as appropriate. No interactions were detected between BMI and u-hCG. Different letters denote difference among groups (^a^ vs. ^b^; P<0.05). BMI; Body mass index, u-hCG; Urinary derived human chorionic gonadotropin, FSH; Follicle-stimulating hormone, AFC; Antral follicle count, and NS; Nonsignificant.

Next, we performed a two-way ANOVA to evaluate the effect of both BMI and u-hCG dose categories on superovulation variables, CPRs and LBRs (Tables2, 3). As expected, a significantly decreased trend existed in serum hCG levels 12-14 hours after u-hCG administration as the strata of BMI increased even though the doses of u-hCG administered did not differ in relation to BMI (Tables2). Analysis of the BMI groups showed that obese patients had significantly more total numbers of developed follicles and numbers of follicles ≥14 mm on the day the u-hCG trigger was administered compared with the other BMI groups (Table 2). 

**Table 2:** Superovulation variables, serum hCG, oocytes and embryo parameters, CPRs and LBRs in relation to BMI categories and u-hCG dosages


Variable		BMI		
	<25 (n=136)	≥25 - <30 (n=84)	≥30 (n=75)	P value

Dose of HCG administered	10547.9 ± 3169.0	10333.3 ± 2784.4	10166.7 ± 2849.2	0.623
Serum hCG (IU) 12-14 hours after hCG trigger	312.7 ± 16.5^a^	230.4 ± 12.0^b^	162.8 ± 9.6^c^	0.001
Gn stimulation (days)	10.2 ± 0.2	10.6 ± 0.2	10.2 ± 0.2	NS
Total Gn dose (IU)	4454.0 ± 286.5	5342.9 ± 404.9.5	4592.9 ± 415.5	NS
Total follicles developed	19.7 ± 0.9^b^	19.4 ± 1.1^b^	22.5 ± 1.1^a^	0.029
Follicles ≥14 mm day of hCG	10.9 ± 0.4^b^	10.6 ± 0.6^b^	12.2 ± 0.6^a^	0.049
E_2_ day of hCG (pg/mL)	2508.7 ± 84.1	2396.3 ± 194.2	2363.5 ± 107.8	NS
Endometrium thickness on day of hCG (mm)	10.2 ± 0.2	10.6 ± 0.2	10.7 ± 0.3	NS
Oocytes collected	13.7 ± 0.7	13.9 ± 1.1	15.4 ± 0.9	NS
Mature oocytes	10.9 ± 0.6	11.4 ± 0.8	12.0 ± 0.7	NS
Oocytes fertilized	8.4 ± 0.5	9.2 ± 0.7	9.3 ± 0.6	NS
Embryos transferred	2.6 ± 0.1	2.5 ± 0.1	2.8 ± 0.1	NS
CPR (%)	61/136 (44.9)	35/84 (41.7)	28/75 (37.3)	NS
LBR (%), n=292*	54/136 (39.7)	30/81 (37.0)	24/75 (32.0)	NS


Except otherwise stated results are means ± SE from two-way ANOVAs with Tukey post hoc analysis as appropriate. No interactions were detected between BMI and hCG. Different letters denote differences among groups (^a^ vs. ^b^, ^a^ vs.^c^, and ^b^ vs. ^c^; P<0.05). BMI; Body mass index, u-hCG; Urinary derived human chorionic gonadotropin, Gn; Gonadotropin, E_2_; Estradiol, CPR; Clinical pregnancy rate, LBR; Live birth rate, NS; Nonsignificant, and *; 3 cycles were lost to follow up hence the outcome of pregnancy in these 3 cases was not known.

However, BMI categories did not impact other superovulation parameters: the number of days Gn were administered and the total dose of Gn used for superovulation; E_2_ levels and endometrial thickness on the day the u-hCG was administered; and the number of oocytes collected, the number of mature and fertilized oocytes, and the number of embryos transferred. 

As expected, there was a significant increased trend in the serum hCG levels at 12-14 hours in relation to the amount of u-hCG given to trigger ovulation even though the BMI did not differ among patients in the three administered doses of u-hCG (Table 3). In addition, on the day we administered the u-hCG trigger, the total number of follicles that developed, follicles ≥14 mm, and serum E_2_ levels were significantly higher in those who received 5000 IU compared with the other two uhCG groups. Those who received 10000 IU had significantly higher E_2_ compared with those who received 15000 IU. We observed similar trends with the number of oocytes collected, number of mature oocytes, and number of fertilized oocytes (Table 3). 

**Table 3:** Superovulation variables, serum hCG, oocytes and embryo parameters, CPRs and LBRs in relation to u-hCG dosages


Variable		u-hCG		
	5000 (n=39)	10000 (n=193)	15000 (n=63)	P value

BMI	27.0 ± 5.7	27.8 ± 6.5	26.0 ± 6.1	0.11
Serum hCG (IU) 12-14 hours after hCG trigger	129.2 ± 10.1^c^	229.2 ± 8.5^b^	394.0 ± 27.0^a^	0.001
Gn. stimulation (days)	10.4 ± 0.3	10.2 ± 0.1	10.6 ± 0.2	NS
Total Gn dose (IU)	4019.6 ± 551.0	5150.1 ± 246.0	5218.5 ± 429.3	NS
Total follicles developed	30.7 ± 1.6^a^	18.6 ± 0.7^b^	19.5 ± 1.2^b^	0.001
Follicles ≥14 mm day of hCG	17.1 ± 0.9^a^	10.2 ± 0.3^b^	10.2 ± 0.6^b^	0.001
E_2_ day of hCG (pg/mL)	3981 ± 88.0^a^	2293.5 ± 57.1^b^	1930 ± 80.9^c^	0.001
Endometrium thickness on day of hCG (mm)	10.7 ± 0.4	10.4 ± 0.2	10.5 ± 0.3	NS
Oocytes collected	22.6 ± 1.6^a^	13.4 ± 0.6^b^	11.4 ± 0.8^b^	0.001
Mature oocytes	18.8 ± 1.0^a^	10.5 ± 0.5^b^	9.0 ± 0.6^b^	0.001
Oocytes fertilized	14.8 ± 0.9^a^	8.2 ± 0.4^b^	7.2 ± 0.5^b^	0.001
Embryos transferred	2.5 ± 0.1	2.7 ± 0.1	2.6 ± 0.1	NS
CPR (%)	17/39 (43.6)	79/193 (40.9)	28/63 (44.4)	NS
LBR (%), n=292*	16/39 (41.0)	67/190 (35.3)	25/63 (39.7)	NS


Except otherwise stated results are means ± SE from two-way ANOVAs with Tukey post hoc analysis as appropriate. No interactions were detected between BMI and hCG. Different letters denote differences among groups (^a^ vs. ^b^, ^a^ vs.^c^, and ^b^ vs. ^c^; P<0.05). BMI; Body mass index, u-hCG; Urinary derived human chorionic gonadotropin, Gn; Gonadotropin, E_2_; Estradiol; CPR; Clinical pregnancy rate, LBR; Live birth rate; NS; Nonsignificant, and *; 3 cycles were lost to follow up hence the outcome of pregnancy in these 3 cases was not known.

With two-way ANOVA, although the CPRs and LBRs were lowest in obese patients, the differences were not statistically significant. CPRs and LBRs were also not influenced by the different doses of u-hCG administered to trigger ovulation. We performed forward stepwise logistic regression in order to determine whether any of the significant differences (age, based on significant correlations with both CPRs and LBRs) seen in the BMI groups and the doses of u-hCG administered influenced the CPRs and LBRs. Only maternal age (P<0.001) influenced CPRs (OR=0.85, CI: 0.79-0.91) and LBRs (OR=0.84, CI: 0.78-0.90). These findings suggested that for a one-unit increase in maternal age, we would expect approximately 15% decrease in the odds of a clinical pregnancy and 16% decrease in the odds of a live birth.

There were 3 patients in the entire cohort who developed moderate to severe OHSS; all were in the subgroup of patients that received 10000 IU of u-hCG for trigger. These patients had a peak E_2_ of 2604, 2662, and 3881 pg/mL. They developed 23- 26 follicles and had 13-20 oocytes collected. All were pregnant. One delivered a singleton and the other two delivered triplets. 

## Discussion

The results of this study have shown the effectiveness of low dose u-hCG compared with higher doses for final oocyte maturation in IVF/ICSI cycles. This finding was associated with similar CPRs and LBRs independent of BMI. We believed these results were valid given that we found no interaction between BMI and the u-hCG categories. Our results, however, concurred with other researchers who found an inverse relationship between serum hCG concentration and BMI (15- 17, 21-23) which might be a volume distribution phenomenon. While a previous study by our group (17) reported on the impact of lower u-hCG doses in obese patients, we did not report on the CPRs. Others (15, 16, 18) reported on the CPRs in a small number of patients (≤50), but did not report on the LBRs. Carrell et al. (24) observed that patients with a BMI >30 had significantly lower follicular fluid hCG levels and CPRs after the administration of 10000 IU of u-hCG compared to patients with BMIs of 20-30 and <20, which suggested that BMI could influence CPRs at a dose of 10000 IU u-hCG. However, they used arbitrary BMI categories that did not conform to the World Health Organization definition. In the current study, we used robust statistical analyses and determined that neither BMI nor the different doses of u-hCG influenced oocyte maturation, CPRs, and LBRs, which indicated that these doses were adequate for successful treatment outcome. 

Drug metabolism and distribution depend on hepatic clearance, rate of excretion, and the volume of adipose tissue (25). Therefore, it is understandable that concerns exist as to the suitability of lower doses of u-hCG in initiating and completing final oocyte maturation in obese patients. The threshold for the lowest levels of serum hCG 12-14 hours after u-hCG administration required for final oocyte maturation (i.e., a serum hCG level when no oocyte would be collected) remains a contentious issue. Initial trials of early hCG formulation (Profasi) have shown that a mean serum level of ≥129 mIU/mL one day after injection sufficiently induced follicular maturation and adequate luteinization (26). This was in keeping with findings in the current study which showed that despite lower levels of serum hCG in those who received 5000 IU u-hCG, forward logistic regression indicated that the number of mature oocytes collected and fertilized, and CPRs and LBRs were not affected. Levy et al. (27) measured serum hCG levels the morning after u-hCG administration and reported that similar numbers of mature oocytes were achieved when cycles were in the lower 5^th^ percentile of serum hCG levels (range: 27-50 mIU/mL) compared to those in the top 5^th^ percentile (range: 300-700 mIU/mL). Stefanis et al. (28) found no correlation between BMI, number of oocytes retrieved, or fertilized and serum concentrations of hCG at 12 and 36 hours following administration of 5000 IU u-hCG. They concluded that neither serum concentrations of hCG nor BMI influenced IVF outcome. They did not categorize BMIs; and used correlation coefficients to assess relationships between hCG levels and BMI, and used fertilization and biochemical pregnancy as their main outcome measures.

## Conclusion

We have concluded that BMI need not be taken into consideration in choosing the appropriate dose of u-hCG to effect final oocyte maturation prior to oocyte collection in IVF as low dose uhCG is equality effective. The common practice of administering lower doses of u-hCG to trigger final oocyte maturation in patients at high risk of OHSS does not compromise CPRs and LBRs irrespective of BMI. Our study could not confirm the need for the use of longer needles to reach the muscles rather than adipose tissue for administration of the lower dose of hCG (5000 IU) in obese women. Only maternal age negatively impacted CPRs and LBRs in the present study, a phenomenon that has been shown to be associated with an age dependent increase in aneuploid embryos (29). The common practice of administering lower doses of u-hCG to trigger final oocyte maturation in patients at high risk of OHSS should not and does not compromise CPRs and LBRs irrespective of BMI.

Finally, this study had several limitations. Our study was not randomized, hence has the inherent limitations of a retrospective study such as incomplete information for some of the patients as can be deduced from the tables. There were 10 patients with BMI 17.3-18.4 included in the <25 BMI group but inclusion of these patients did not influence the results of this study. The overall sample size was small (n=295) and could have influenced the findings. An adequately powered multicenter randomized study would confirm the current study findings; however, such a study might not be feasible because of the sample size. In the absence of results from randomized studies, management of obese patients that undergo ART will likely need to be based on results of observational studies such as the current study. 

## References

[1] Davies MJ (2006). Evidence for effects of weight on reproduction in women. Reprod Biomed Online.

[2] Rowland AS, Baird DD, Long S, Wegienka G, Harlow SD, Alavanja M (2002). Influence of medical conditions and lifestyle factors on the menstrual cycle. Epidemiol- ogy.

[3] Jensen TK, Scheike T, Keiding N, Schaumburg I, Grand- jean P (1999). Fecundability in relation to body mass and men- strual cycle patterns. Epidemiology.

[4] Jacobsen BK, Knutsen SF, Oda K, Fraser GE (2012). Obesity at age 20 and the risk of miscarriages, irregular periods and reported problems of becoming pregnant: the adventist health study-2. Eur J Epidemiol.

[5] Practice Committee of American Society for Reproduc- tive Medicine (2008). Obesity and reproduction: an educational bulletin. Fertil Steril.

[6] Moley KH, Chi MM, Knudson CM, Korsmeyer SJ, Mueck- ler MM (1998). Hyperglycemia induces apoptosis in pre-implan- tation embryos through cell death effector pathways. Nat Med.

[7] Sutton-McDowall ML, Gilchrist RB, Thompson JG (2010). The pivotal role of glucose metabolism in determining oo- cyte developmental competence. Reproduction.

[8] Igosheva N, Abramov AY, Poston L, Eckert JJ, Fleming TP, Duchen MR (2010). Maternal diet-induced obesity al- ters mitochondrial activity and redox status in mouse oo- cytes and zygotes. PLoS One.

[9] Luke B, Brown MB, Stern JE, Missmer SA, Fujimoto VY, Leach R (2011). Female obesity adversely affects assisted reproductive technology (ART) pregnancy and live birth rates. Hum Reprod.

[10] Bellver J, Pellicer A, García-Velasco JA, Ballesteros A, Remohí J, Meseguer M (2013). Obesity reduces uterine recep- tivity: clinical experience from 9,587 first cycles of ovum donation with normal weight donors. Fertil Steril.

[11] Metwally M, Tuckerman EM, Laird SM, Ledger WL, Li TC (2007). Impact of high body mass index on endometrial mor- phology and function in the peri-implantation period in women with recurrent miscarriage. Reprod Biomed On- line.

[12] Robker RL, Akison LK, Bennett BD, Thrupp PN, Chura LR, Russell DL (2009). Obese women exhibit differences in ovarian metabolites, hormones, and gene expression compared with moderate-weight women. J Clin Endo- crinol Metab.

[13] Rabinson J, Meltcer S, Zohav E, Gemer O, Anteby EY, Orvieto R (2008). GnRH agonist versus GnRH antagonist in ovarian stimulation: the influence of body mass index on in vitro fertilization outcome. Fertil Steril.

[14] Tu J, Lin G, Lu C, Gong F (2014). A novel modified ultra-long ag- onist protocol improves the outcome of high body mass index women with polycystic ovary syndrome undergo- ing IVF/ICSI. Gynecol Endocrinol.

[15] Salha O, Dada T, Sharma V (2001). Influence of body mass index and self-administration of hCG on the outcome of IVF cycles: a prospective cohort study. Hum Fertil (Camb).

[16] Elkind-Hirsch KE, Bello S, Esparcia L, Phillips K, Sheiko A, McNichol M (2001). Serum human chorionic gonadotropin levels are correlated with body mass index rather than route of administration in women undergoing in vitro fertilization--embryo transfer using human menopausal gonadotropin and intracytoplasmic sperm injection. Fertil Steril.

[17] Detti L, Mitwally MF, Rode A, Yelian FD, Kruger M, Dia- mond MP (2007). Serum human chorionic gonadotropin level after ovulation triggering is influenced by the pa- tient's body mass index and the number of larger folli- cles. Fertil Steril.

[18] Sills ES, Drews CD, Perloe M, Kaplan CR, Tucker MJ (2001). Periovulatory serum human chorionic gonadotropin (hCG) concentrations following subcutaneous and in- tramuscular nonrecombinant hCG use during ovulation induction: a prospective, randomized trial. Fertil Steril.

[19] Kolibianakis EM, Papanikolaou EG, Tournaye H, Camus M, Van Steirteghem AC, Devroey P (2007). Triggering final oo- cyte maturation using different doses of human chorionic gonadotropin: a randomized pilot study in patients with polycystic ovary syndrome treated with gonadotropin- releasing hormone antagonists and recombinant follicle- stimulating hormone. Fertil Steril.

[20] Tsoumpou I, Muglu J, Gelbaya TA, Nardo LG (2009). Symposium: update on prediction and management of OHSS.Optimal dose of HCG for final oocyte maturation in IVF cycles: absence of evidence?. Reprod Biomed Online.

[21] Chan CC, Ng EH, Chan MM, Tang OS, Lau EY, Yeung WS (2003). Bioavailability of hCG after intramuscular or subcutaneous injection in obese and non-obese women. Hum Reprod.

[22] Matorras R, Meabe A, Mendoza R, Prieto B, Ramón O, Mugica J (2012). Human chorionic gonadotropin (hCG) plasma levels at oocyte retrieval and IVF outcomes. J Assist Reprod Genet.

[23] Shah DK, Missmer SA, Correia KF, Ginsburg ES (2014). Phar- macokinetics of human chorionic gonadotropin injection in obese and normal-weight women. J Clin Endocrinol Metab.

[24] Carrell DT, Jones KP, Peterson CM, Aoki V, Emery BR, Campbell BR (2001). Body mass index is inversely related to in- trafollicular HCG concentrations, embryo quality and IVF outcome. Reprod Biomed Online.

[25] Hanley MJ, Abernethy DR, Greenblatt DJ (2010). Effect of obe- sity on the pharmacokinetics of drugs in humans. Clin Pharmacokinet.

[26] Driscoll GL, Tyler JP, Hangan JT, Fisher PR, Birdsall MA, Knight DC (2000). A prospective, randomized, controlled, double-blind, double-dummy comparison of recombinant and urinary HCG for inducing oocyte maturation and fol- licular luteinization in ovarian stimulation. Hum Reprod.

[27] Levy G, Hill MJ, Ramirez C, Plowden T, Pilgrim J, Howard RS (2013). Serum human chorionic gonadotropin levels on the day before oocyte retrieval do not correlate with oo- cyte maturity. Fertil Steril.

[28] Stefanis P, Das S, Barsoum-Derias E, Kingsland C, Lew- is-Jones I, Gazvani R (2007). Relationship between serum hu- man chorionic gonadotrophin levels and body mass in- dex in women undergoing in vitro fertilisation cycles. Eur J Obstet Gynecol Reprod Biol.

[29] Munné S, Alikani M, Tomkin G, Grifo J, Cohen J (1995). Embryo morphology, developmental rates, and maternal age are correlated with chromosome abnormalities. Fertil Steril.

